# *catena*-Poly[[[aqua­(formato-κ*O*)(1,10-phenanthroline-κ^2^ *N*,*N*′)manganese(II)]-μ-formato-κ^2^ *O*:*O*′] monohydrate]

**DOI:** 10.1107/S1600536811020575

**Published:** 2011-06-11

**Authors:** Wei Xu

**Affiliations:** a Center of Applied Solid State Chemistry Research, Ningbo University, Ningbo 315211, People’s Republic of China

## Abstract

The title compound, {[Mn(HCOO)_2_(C_12_H_8_N_2_)(H_2_O)]·H_2_O}_*n*_, consists of polymeric chains of the complex [Mn(HCOO)_2_(phen)(H_2_O)]_∞_ (phen is 1,10-phenanthroline) with solvent water mol­ecules. The chains contain six-coordinate Mn^II^ ions bridged by formate anions. They are further extended into a three-dimensional network *via* O—H⋯O hydrogen-bonding inter­actions and inter­chain π–π stacking inter­actions, with a centroid–centroid distance of 3.679 (4) Å.

## Related literature

For the design and synthesis of coordination polymer complexes and their potential applications, see: Robin & Fromm (2006 ▶); Farrusseng *et al.* (2008 ▶); Chen *et al.* (2010 ▶). For the formate anion as a ligand, see: Yuan *et al.* (2008 ▶); Hagen *et al.* (2009 ▶); Hu *et al.* (2009 ▶); Paredes-Gaecía (2009 ▶). For a related structure, see: Janiak (2000 ▶). 
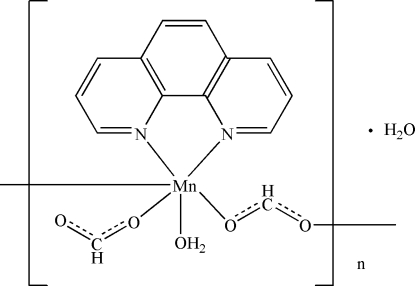


## Experimental

### 

#### Crystal data

[Mn(HCO_2_)_2_(C_12_H_8_N_2_)(H_2_O)]·H_2_O
*M* *_r_* = 361.21
Orthorhombic, 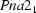

*a* = 19.260 (4) Å
*b* = 12.161 (2) Å
*c* = 6.5493 (13) Å
*V* = 1534.0 (5) Å^3^
*Z* = 4
Mo *K*α radiation
μ = 0.89 mm^−1^
*T* = 295 K
0.31 × 0.12 × 0.09 mm

#### Data collection

Rigaku R-AXIS RAPID diffractometer
Absorption correction: multi-scan (*ABSCOR*; Higashi, 1995 ▶) *T*_min_ = 0.664, *T*_max_ = 0.791
11493 measured reflections
2644 independent reflections
1921 reflections with *I* > 2σ(*I*)
*R*_int_ = 0.047

#### Refinement

*R*[*F* ^2^ > 2σ(*F* ^2^)] = 0.037
*wR*(*F* ^2^) = 0.111
*S* = 1.20
2644 reflections
209 parameters
1 restraint
H-atom parameters constrained
Δρ_max_ = 0.70 e Å^−3^
Δρ_min_ = −0.93 e Å^−3^
Absolute structure: Flack (1983 ▶), 1165 Friedel pairs
Flack parameter: 0.01 (4)

### 

Data collection: *RAPID-AUTO* (Rigaku, 1998 ▶); cell refinement: *RAPID-AUTO*; data reduction: *CrystalStructure* (Rigaku/MSC, 2004 ▶); program(s) used to solve structure: *SHELXS97* (Sheldrick, 2008 ▶); program(s) used to refine structure: *SHELXL97* (Sheldrick, 2008 ▶); molecular graphics: *SHELXTL* (Sheldrick, 2008 ▶); software used to prepare material for publication: *SHELXL97*.

## Supplementary Material

Crystal structure: contains datablock(s) global, I. DOI: 10.1107/S1600536811020575/fj2422sup1.cif

Structure factors: contains datablock(s) I. DOI: 10.1107/S1600536811020575/fj2422Isup2.hkl

Additional supplementary materials:  crystallographic information; 3D view; checkCIF report

## Figures and Tables

**Table 1:** Hydrogen-bond geometry (Å, °)

*D*—H⋯*A*	*D*—H	H⋯*A*	*D*⋯*A*	*D*—H⋯*A*
O5—H5*B*⋯O2	0.83	1.96	2.713 (5)	150
O5—H5*C*⋯O6	0.85	1.76	2.601 (6)	177
O6—H6*B*⋯O4^i^	0.83	1.88	2.693 (8)	166
O6—H6*C*⋯O4^ii^	0.83	2.13	2.864 (9)	145

Symmetry codes: (i) 
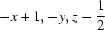
; (ii) 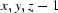.

**Fig. 1.**
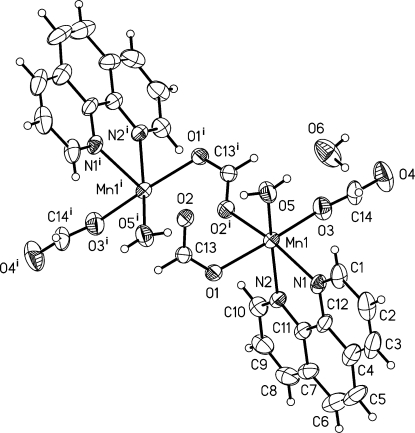
ORTEP view of the title compound (40% thermal ellipsoids) showing the atom-labeling scheme. [Symmetry Code: (i) 1-x, 1-y, 1/2+z)

**Fig. 2.**
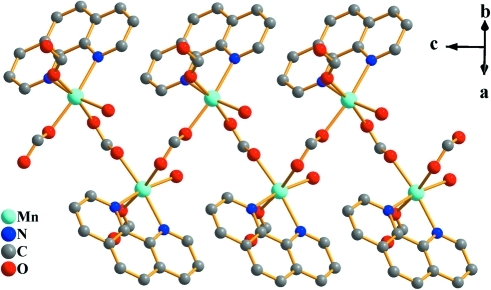
one dimensional zigzag chain along [001]

**Fig. 3.**
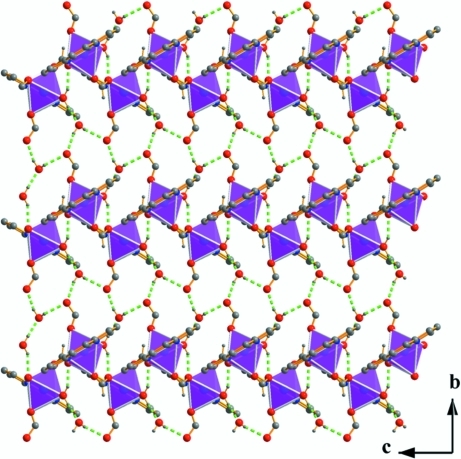
A view of a single layer of I, hydrogen-bonding is indicated as dashed lines.

## supplementary crystallographic information

### Comment

In recent years, extensive efforts have been dedicated to the design and construction of coordination polymers because their supramolecular architectures with specific topologies may endow them with promising properties for material chemistry, such as gas sorption, storage and separations, molecular recognition, heterogeneous catalysis, nonlinear optics and magnetic properties (Robin & Fromm, 2006; Farrusseng, *et al.*, 2008; Chen, *et al.*, 2010). Investigations on a series of transition metal formate anions showed that it tend to function as a bidentate ligand to bridge metal atoms into one-dimensional chains, two-dimensional layers and three-dimensional networks (Hagen, *et al.*, 2009; Hu, *et al.*, 2009; Paredes-Gaecía, 2009). In the present contribution, we report a new manganese(II) complex, [Mn(HCOO)_2_(phen)(H_2_O)].H_2_O (I), resulting from self-assembly of Mn^2+^ ions, 1,10-phenanthroline and formic acid. It is isostructural with the previously reported [Co(HCOO)_2_(phen)(H_2_O)].H_2_O complex (Yuan, *et al.*, 2008).

Compound I consists of an neutral one-dimensional zigzag chains [Mn(HCOO)_2_(phen)(H_2_O)]_n_ and lattice water molecules. As shown in Fig. 1, each Mn atom is octahedral coordination by two N atoms of phen ligand, two O atoms of two bridging formate anions, one O atom of one terminal formate anion and one O atom of the coordination water molecule. The octehedral coordination around the Mn atoms are strongly distorted since the diametrical and non-diametrical bond angles indicate significant deviations from 180° and 90°, respectively. The Mn-O distances are in the range of 2.134 (5)-2.228 (4) Å, while the Mn-N distances are 2.246 (5) and 2.295 (5) Å. Then two neighboring Mn^II^ centers connected by formate anion with the distance of 5.474 (5) Å form one-dimensional zigzag chain along [001] (Fig. 2).

The coordinated water molecule forms a strong intra-chain hydrogen bond to the carboxyl O2 with d(O···O) = 2.713 (5) Å and <O-H···O = 150°. There are three kinds of independent inter-chain hydrogen bonds responsible for the two-dimensional layers assembly (Fig. 3, Table 1). One kind of the inter-chain O-H···O hydrogen bonds is formed between the O-H group of coordinated water molecules acting as acceptors (the O···O distance is 2.601 (6) Å with a O-H···O angle of 177°). The other two kinds are formed between the O-H groups of uncoordinated water molecules and the uncoordinated oxygen atoms of the carboxyl groups from the coordianted terminal formate anions in two adjacent chains, with the different O···O distances of 2.693 (8) and 2.864 (9) Å, and two different O-H···O angles of 166° and 145°, respectively. The phen ligands chelating Mn atoms exhibit nearly perfect coplanarity. Two neighboring phen ligands of different chains parallelly face opposite directions at an interplanar centroid to centroid distance of 3.679 (4) Å, with the quinoline fragments partially covered, which suggests significant inter-chain π-π stacking interactions (Janiak, 2000). Acoording to the above description, it is clear that the π-π interactions and inter-chain hydrogen bonding interactions are responsible for the supramolecular assembly of the three-dimensional network.

### Experimental

Addition of 2.0 mL (1.0 M) NaOH to a stirred aqueous of 0.201 g (1.0 mmol) MnCl_2_.4H_2_O in 5.0 mL H_2_O yield yellowish precipitate, which was then separated by centrifugation, followed by washing with double-distilled water until no detectable Cl^-^ anions in supernatant. The precipitate was added to a stirred ethanolic aqueous solution of 0.198 g (1.0 mmol) 1,10-phenanthroline monohydrate in 20 mL EtOH/H_2_O (v:v = 1: 1). To the mixture was added 2.0 mL (1.0 M) HCOOH and the yellowish suspension was further stirred for ca. 30 min. After filtration, the solution (pH = 6.58) was allowed to stand at room temperature. Slow evaporation for two weeks affored yellowish crystals (yield 62% based on the initial MnCl_2_.4H_2_O input).

### Refinement

All H atoms bound to C were position geometrically and refined as riding, with C-H = 0.93 Å and *U*_iso_(H) = 1.2*U*_eq_(C). H atoms attached to O were located in difference Fourier maps and placed at fixed positions with *U*_iso_(H) = 1.5*U*_eq_(O).

### Crystal data

**Table:**

[Mn(HCO_2_)_2_(C_12_H_8_N_2_)(H_2_O)]·H_2_O	*F*(000) = 740
*M**_r_* = 361.21	*D*_x_ = 1.564 Mg m^−^^3^
Orthorhombic, *P**n**a*2_1_	Mo *K*α radiation, λ = 0.71073 Å
Hall symbol: P 2c -2n	Cell parameters from 8376 reflections
*a* = 19.260 (4) Å	θ = 3.4–27.4°
*b* = 12.161 (2) Å	µ = 0.89 mm^−^^1^
*c* = 6.5493 (13) Å	*T* = 295 K
*V* = 1534.0 (5) Å^3^	Needle, yellow
*Z* = 4	0.31 × 0.12 × 0.09 mm

### Data collection

**Table:**

Rigaku R-AXIS RAPID diffractometer	2644 independent reflections
Radiation source: fine-focus sealed tube	1921 reflections with *I* > 2σ(*I*)
graphite	*R*_int_ = 0.047
ω scans	θ_max_ = 25.0°, θ_min_ = 3.4°
Absorption correction: multi-scan (*ABSCOR*; Higashi, 1995)	*h* = −22→22
*T*_min_ = 0.664, *T*_max_ = 0.791	*k* = −14→14
11493 measured reflections	*l* = −7→7

### Refinement

**Table:**

Refinement on *F*^2^	Hydrogen site location: inferred from neighbouring sites
Least-squares matrix: full	H-atom parameters constrained
*R*[*F*^2^ > 2σ(*F*^2^)] = 0.037	*w* = 1/[σ^2^(*F*_o_^2^) + (0.0135*P*)^2^ + 2.7605*P*] where *P* = (*F*_o_^2^ + 2*F*_c_^2^)/3
*wR*(*F*^2^) = 0.111	(Δ/σ)_max_ < 0.001
*S* = 1.20	Δρ_max_ = 0.70 e Å^−^^3^
2644 reflections	Δρ_min_ = −0.93 e Å^−^^3^
209 parameters	Extinction correction: *SHELXL97* (Sheldrick, 2008), Fc^*^=kFc[1+0.001xFc^2^λ^3^/sin(2θ)]^-1/4^
1 restraint	Extinction coefficient: 0.0025 (6)
Primary atom site location: structure-invariant direct methods	Absolute structure: Flack (1983), 1165 Friedel pairs
Secondary atom site location: difference Fourier map	Flack parameter: 0.01 (4)

### Special details

**Table:**

Geometry. All esds (except the esd in the dihedral angle between two l.s. planes) are estimated using the full covariance matrix. The cell esds are taken into account individually in the estimation of esds in distances, angles and torsion angles; correlations between esds in cell parameters are only used when they are defined by crystal symmetry. An approximate (isotropic) treatment of cell esds is used for estimating esds involving l.s. planes.
Refinement. Refinement of F^2^ against ALL reflections. The weighted R-factor wR and goodness of fit S are based on F^2^, conventional R-factors R are based on F, with F set to zero for negative F^2^. The threshold expression of F^2^ > 2σ(F^2^) is used only for calculating R-factors(gt) etc. and is not relevant to the choice of reflections for refinement. R-factors based on F^2^ are statistically about twice as large as those based on F, and R- factors based on ALL data will be even larger.

### Fractional atomic coordinates and isotropic or equivalent isotropic displacement parameters (Å^2^)

**Table:**

	*x*	*y*	*z*	*U*_iso_*/*U*_eq_	
Mn1	0.57992 (4)	0.37155 (6)	0.67916 (16)	0.0454 (3)	
N1	0.6768 (3)	0.3026 (4)	0.5202 (9)	0.0525 (13)	
N2	0.6704 (2)	0.4167 (4)	0.8785 (8)	0.0470 (12)	
C1	0.6796 (4)	0.2444 (5)	0.3480 (10)	0.068 (2)	
H1A	0.6385	0.2277	0.2802	0.082*	
C2	0.7429 (5)	0.2076 (6)	0.2658 (13)	0.087 (3)	
H2A	0.7435	0.1662	0.1464	0.105*	
C3	0.8034 (5)	0.2333 (7)	0.3629 (15)	0.091 (3)	
H3A	0.8455	0.2099	0.3084	0.110*	
C4	0.8027 (4)	0.2946 (6)	0.5440 (13)	0.073 (2)	
C5	0.8643 (4)	0.3266 (7)	0.6559 (18)	0.093 (3)	
H5A	0.9077	0.3074	0.6049	0.112*	
C6	0.8603 (4)	0.3828 (8)	0.8299 (16)	0.096 (3)	
H6A	0.9010	0.4006	0.8988	0.116*	
C7	0.7958 (3)	0.4161 (6)	0.9127 (12)	0.067 (2)	
C8	0.7882 (4)	0.4722 (7)	1.0989 (12)	0.079 (3)	
H8A	0.8274	0.4906	1.1742	0.095*	
C9	0.7246 (4)	0.4999 (5)	1.1701 (14)	0.0725 (19)	
H9A	0.7197	0.5372	1.2932	0.087*	
C10	0.6668 (4)	0.4714 (5)	1.0556 (11)	0.0600 (17)	
H10A	0.6233	0.4913	1.1043	0.072*	
C11	0.7335 (3)	0.3880 (5)	0.8082 (10)	0.0513 (16)	
C12	0.7379 (3)	0.3280 (5)	0.6200 (10)	0.0553 (19)	
C13	0.5493 (3)	0.5910 (5)	0.4494 (9)	0.0508 (15)	
H13	0.5562	0.6662	0.4325	0.061*	
O1	0.5939 (2)	0.5392 (3)	0.5480 (7)	0.0537 (11)	
O2	0.4966 (2)	0.5501 (3)	0.3718 (7)	0.0599 (12)	
C14	0.5604 (4)	0.1288 (6)	0.7827 (12)	0.071 (2)	
H14	0.5642	0.1173	0.6428	0.086*	
O3	0.5629 (3)	0.2210 (4)	0.8388 (7)	0.0747 (14)	
O4	0.5534 (4)	0.0457 (4)	0.8904 (10)	0.112 (2)	
O5	0.5090 (2)	0.3307 (3)	0.4372 (7)	0.0719 (14)	
H5B	0.4990	0.3890	0.3784	0.108*	
H5C	0.5014	0.2710	0.3749	0.108*	
O6	0.4906 (3)	0.1479 (4)	0.2384 (9)	0.115 (2)	
H6B	0.4780	0.0927	0.3042	0.173*	
H6C	0.5237	0.1210	0.1706	0.173*	

### Atomic displacement parameters (Å^2^)

**Table:**

	*U*^11^	*U*^22^	*U*^33^	*U*^12^	*U*^13^	*U*^23^
Mn1	0.0384 (4)	0.0469 (4)	0.0509 (5)	0.0040 (4)	0.0029 (6)	−0.0014 (6)
N1	0.052 (3)	0.052 (3)	0.054 (3)	0.013 (2)	0.016 (3)	0.008 (3)
N2	0.039 (3)	0.053 (3)	0.049 (3)	0.005 (2)	0.002 (2)	0.004 (3)
C1	0.094 (6)	0.053 (4)	0.056 (4)	0.020 (4)	0.035 (4)	−0.002 (4)
C2	0.129 (8)	0.061 (4)	0.073 (5)	0.023 (5)	0.050 (6)	0.012 (4)
C3	0.096 (7)	0.075 (6)	0.103 (7)	0.043 (5)	0.049 (6)	0.036 (6)
C4	0.062 (5)	0.080 (5)	0.078 (5)	0.021 (4)	0.030 (4)	0.041 (4)
C5	0.039 (4)	0.129 (7)	0.112 (8)	0.019 (4)	0.019 (5)	0.063 (8)
C6	0.050 (5)	0.133 (9)	0.106 (8)	−0.001 (5)	−0.002 (5)	0.059 (7)
C7	0.043 (4)	0.078 (5)	0.078 (6)	−0.007 (4)	−0.011 (4)	0.040 (4)
C8	0.075 (5)	0.083 (5)	0.081 (6)	−0.026 (4)	−0.036 (4)	0.033 (4)
C9	0.082 (5)	0.069 (4)	0.066 (4)	−0.018 (4)	−0.029 (5)	0.013 (5)
C10	0.069 (5)	0.058 (4)	0.053 (4)	−0.001 (4)	−0.005 (4)	0.005 (3)
C11	0.045 (4)	0.057 (4)	0.051 (4)	0.005 (3)	0.003 (3)	0.023 (3)
C12	0.040 (4)	0.057 (4)	0.068 (5)	0.017 (3)	0.011 (3)	0.024 (3)
C13	0.047 (4)	0.047 (4)	0.058 (4)	0.000 (3)	−0.007 (3)	0.013 (3)
O1	0.047 (2)	0.049 (2)	0.065 (3)	−0.0018 (19)	−0.016 (2)	0.008 (2)
O2	0.050 (3)	0.052 (3)	0.078 (3)	0.004 (2)	−0.019 (2)	0.008 (2)
C14	0.090 (6)	0.048 (4)	0.076 (5)	0.019 (4)	0.024 (4)	0.015 (4)
O3	0.090 (4)	0.058 (3)	0.075 (4)	0.005 (3)	0.021 (3)	0.002 (3)
O4	0.164 (6)	0.056 (3)	0.117 (5)	0.008 (4)	0.023 (5)	0.024 (4)
O5	0.085 (4)	0.048 (3)	0.082 (3)	0.000 (2)	−0.032 (3)	0.001 (2)
O6	0.165 (6)	0.085 (4)	0.095 (5)	−0.030 (4)	0.028 (4)	−0.033 (3)

### Geometric parameters (Å, °)

**Table:**

Mn1—O3	2.134 (5)	C6—H6A	0.9300
Mn1—O5	2.150 (4)	C7—C8	1.405 (11)
Mn1—O2^i^	2.161 (4)	C7—C11	1.422 (9)
Mn1—O1	2.228 (4)	C8—C9	1.353 (10)
Mn1—N2	2.246 (5)	C8—H8A	0.9300
Mn1—N1	2.295 (5)	C9—C10	1.385 (9)
N1—C1	1.333 (8)	C9—H9A	0.9300
N1—C12	1.382 (8)	C10—H10A	0.9300
N2—C10	1.339 (9)	C11—C12	1.435 (9)
N2—C11	1.346 (7)	C13—O2	1.240 (7)
C1—C2	1.406 (10)	C13—O1	1.245 (7)
C1—H1A	0.9300	C13—H13	0.9300
C2—C3	1.364 (11)	O2—Mn1^ii^	2.161 (4)
C2—H2A	0.9300	C14—O3	1.180 (8)
C3—C4	1.401 (12)	C14—O4	1.240 (8)
C3—H3A	0.9300	C14—H14	0.9300
C4—C12	1.403 (9)	O5—H5B	0.8290
C4—C5	1.448 (12)	O5—H5C	0.8460
C5—C6	1.331 (13)	O6—H6B	0.8339
C5—H5A	0.9300	O6—H6C	0.8420
C6—C7	1.414 (11)		
			
O3—Mn1—O5	93.75 (19)	C4—C5—H5A	119.2
O3—Mn1—O2^i^	89.28 (17)	C5—C6—C7	121.8 (9)
O5—Mn1—O2^i^	95.71 (18)	C5—C6—H6A	119.1
O3—Mn1—O1	172.92 (19)	C7—C6—H6A	119.1
O5—Mn1—O1	90.21 (16)	C8—C7—C6	124.3 (8)
O2^i^—Mn1—O1	84.49 (17)	C8—C7—C11	116.5 (7)
O3—Mn1—N2	92.55 (19)	C6—C7—C11	119.1 (8)
O5—Mn1—N2	167.9 (2)	C9—C8—C7	121.0 (7)
O2^i^—Mn1—N2	94.70 (18)	C9—C8—H8A	119.5
O1—Mn1—N2	84.64 (16)	C7—C8—H8A	119.5
O3—Mn1—N1	91.92 (18)	C8—C9—C10	118.6 (8)
O5—Mn1—N1	95.6 (2)	C8—C9—H9A	120.7
O2^i^—Mn1—N1	168.5 (2)	C10—C9—H9A	120.7
O1—Mn1—N1	93.53 (17)	N2—C10—C9	123.5 (7)
N2—Mn1—N1	73.86 (18)	N2—C10—H10A	118.3
C1—N1—C12	119.0 (6)	C9—C10—H10A	118.3
C1—N1—Mn1	127.6 (5)	N2—C11—C7	122.3 (7)
C12—N1—Mn1	113.4 (4)	N2—C11—C12	118.6 (6)
C10—N2—C11	118.2 (6)	C7—C11—C12	119.0 (6)
C10—N2—Mn1	125.8 (4)	N1—C12—C4	121.7 (7)
C11—N2—Mn1	116.0 (4)	N1—C12—C11	118.0 (6)
N1—C1—C2	121.9 (8)	C4—C12—C11	120.3 (7)
N1—C1—H1A	119.1	O2—C13—O1	125.0 (6)
C2—C1—H1A	119.1	O2—C13—H13	117.5
C3—C2—C1	119.3 (8)	O1—C13—H13	117.5
C3—C2—H2A	120.3	C13—O1—Mn1	125.5 (4)
C1—C2—H2A	120.3	C13—O2—Mn1^ii^	128.4 (4)
C2—C3—C4	120.5 (8)	O3—C14—O4	127.0 (8)
C2—C3—H3A	119.7	O3—C14—H14	116.5
C4—C3—H3A	119.7	O4—C14—H14	116.5
C3—C4—C12	117.6 (8)	C14—O3—Mn1	131.9 (5)
C3—C4—C5	124.3 (8)	Mn1—O5—H5B	107.1
C12—C4—C5	118.1 (8)	Mn1—O5—H5C	131.6
C6—C5—C4	121.6 (8)	H5B—O5—H5C	118.0
C6—C5—H5A	119.2	H6B—O6—H6C	100.5

Symmetry codes: (i) −*x*+1, −*y*+1, *z*+1/2; (ii) −*x*+1, −*y*+1, *z*−1/2.

### Hydrogen-bond geometry (Å, °)

**Table:**

*D*—H···*A*	*D*—H	H···*A*	*D*···*A*	*D*—H···*A*
O5—H5B···O2	0.83	1.96	2.713 (5)	150
O5—H5C···O6	0.85	1.76	2.601 (6)	177
O6—H6B···O4^iii^	0.83	1.88	2.693 (8)	166
O6—H6C···O4^iv^	0.83	2.13	2.864 (9)	145

Symmetry codes:  (iii) −*x*+1, −*y*, *z*−1/2; (iv) *x*, *y*, *z*−1.
